# Free energy calculations of the functional selectivity of 5-HT_2B_ G protein-coupled receptor

**DOI:** 10.1371/journal.pone.0243313

**Published:** 2020-12-09

**Authors:** Brandon L. Peters, Jinxia Deng, Andrew L. Ferguson

**Affiliations:** 1 Pritzker School of Molecular Engineering, University of Chicago, Chicago, Illinois, United States of America; 2 Zoetis Inc, Kalamazoo, Michigan, United States of America; Universitat Politecnica de Catalunya, SPAIN

## Abstract

G Protein-Coupled Receptors (GPCRs) mediate intracellular signaling in response to extracellular ligand binding and are the target of one-third of approved drugs. Ligand binding modulates the GPCR molecular free energy landscape by preferentially stabilizing active or inactive conformations that dictate intracellular protein recruitment and downstream signaling. We perform enhanced sampling molecular dynamics simulations to recover the free energy surfaces of a thermostable mutant of the GPCR serotonin receptor 5-HT_2B_ in the unliganded form and bound to a lysergic acid diethylamide (LSD) agonist and lisuride antagonist. LSD binding imparts a ∼110 kJ/mol driving force for conformational rearrangement into an active state. The lisuride-bound form is structurally similar to the apo form and only ∼24 kJ/mol more stable. This work quantifies ligand-induced conformational specificity and functional selectivity of 5-HT_2B_ and presents a platform for high-throughput virtual screening of ligands and rational engineering of the ligand-bound molecular free energy landscape.

## Introduction

G Protein-Coupled Receptors (GPCRs), also called seven-pass-transmembrane domain receptors, are a large family of membrane-bound eukaryotic proteins. These proteins undergo conformational change in response to the binding of extracellular drugs or ligands that results in receptor activation, intracellular G-protein recruitment, and downstream signaling [1–3]. GPCRs share a common structure comprising seven transmembrane helices, three extracellular and three intracellular loops, and an extracellular N-terminus and intracellular C-terminus [1]. There is broad pharmaceutical interest in GPCRs due to their importance as targets for therapeutic drugs in the treatment of conditions such as hypertension, schizophrenia, depression, obesity, and Alzheimer’s disease [4]. GPCRs constitute the target of approximately 34% of Food and Drug Administration (FDA)-approved drugs with an annual market of over US$150 billion [4].

Crystallographic structures of a number of GPCRs, including rhodopsin [5, 6], *β*_2_-adrenergic receptor (*β*_2_AR) [7–9], and serotonin receptor 2B (5-hydroxytryptamine receptor 2B, 5-HT_2B_) [10–12], have provided new understanding of ligand and drug binding and the structures of active and inactive states. Molecular simulations have also played an important role in revealing and understanding metastable intermediate states, activation pathways and mechanisms, allostery, and ligand entry and binding by employing a number of unbiased and biased sampling techniques [1, 2, 13–30]. A particularly interesting aspect of ligand binding that has been revealed by molecular simulation is their influence on the GPCR free energy surface [16, 31]. Provasi *et al*. employed metadynamics [32–35] and adiabatic biased molecular dynamics [36] to determine the conformational free energy surface of *β*_2_AR bound with six different ligands with differential physiological responses [19]. These calculations shed light on the molecular root of the ligand-induced conformational specificity through the preferential stabilization of particular *β*_2_AR conformations on the free energy surface of the ligand-receptor complex, with inverse agonists tending to stabilize an inactive state of the receptor and agonists stabilizing the active state. These results present a molecular-level rationalization for “functional selectivity” or “biased signaling” wherein particular ligands stabilize particular GPCR conformations that are predisposed to bind specific intracellular partner proteins and therefore activate particular downstream signaling cascades [12, 19]. This analysis presents a potential avenue for principled drug design by rationally engineering the ligand-bound free energy landscape to stabilize particular conformational states [19].

**Fig 1:**
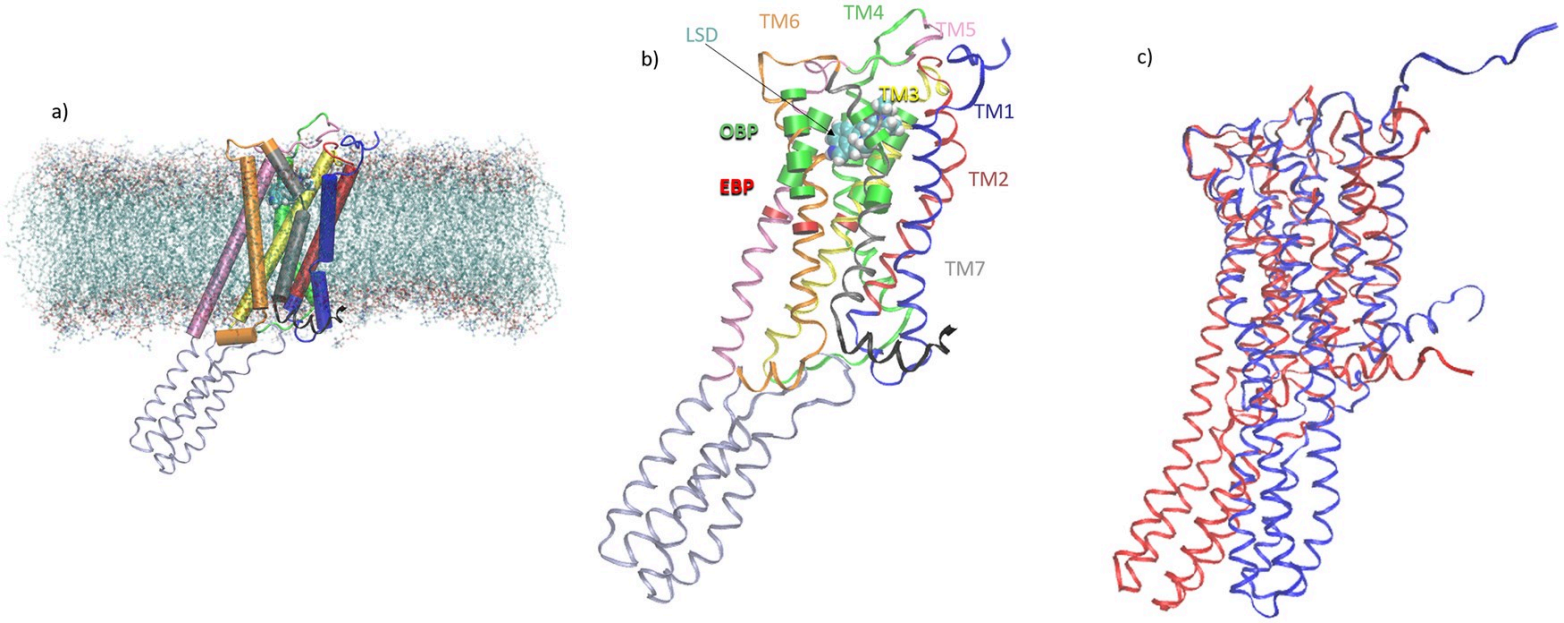
Molecular renderings of 5-HT_2B_-TM GPCR. a) A snapshot of 5-HT_2B_-TM in a membrane-bound state from LSD-bound crystal structure (PDB: 5TVN) reported in Ref. [11]. Water and ions are omitted for clarity. An image of the arrangement of TM1-7 within the membrane. Each helix is represented as a cylinder and employs the same color coding as in panel b) Structure of the LSD-complex, where the transmembrane helices TM1-7 are labeled by color, the orthosteric binding pocket (OBP) and extended binding pocket (EBP) are represented as cartoon ribbons, and the LSD molecule within the binding pocket represented as van der Waals space-filling spheres. c) Superposition of aligned active (red) and inactive (blue) states of 5-HT_2B_-TM. These states correspond to the conformations residing at the bottom of the global free energy minima in the conformational free energy landscapes computed from well-tempered metadynamics simulations (*vide infra*). The active state is distinguished by a large conformational rearrangement of transmembrane helices TM5-7.

In this work, we follow a similar approach to Provasi *et al*. [19] to determine perturbations to the conformational free energy surfaces of a 5-HT_2B_ mutant in response to ligand binding. 5-HT_2B_ is a class A Rhodopsin-like GPCR [37] that mediates the central and peripheral physiologic functions of serotonin [37] (Fig 1a). Impaired function of this receptor has been implicated in fibrosis, cardiovascular and central nervous system disorders, and in cancer [37]. 5-HT_2B_ ligands bind within the orthosteric binding pocket (OBP) and/or extended binding pocket (EBP) [10]. Agonist binding activates the receptor by stabilizing an active state characterized by a large-scale conformational rearrangement of transmembrane helices TM5-7 (Fig 1b–1d) that protrude into the intracellular environment [1, 31] (Fig 1d). This conformational change induces downstream intracellular signaling events, typically by promoting the binding of a cellular partner protein such as G-protein or arrestin [1, 10].

Experimental crystal structures and structure-directed mutagenesis experiments conducted on 5-HT_2B_ by McCorvy *et al*. [10] showed that the differential binding poses of lysergic acid diethylamide (LSD)—a prototypical agonist—and lisuride—a prototypical antagonist that differs from LSD only in its stereochemistry and two extra atoms in a NH group—were responsible for the different pharmacological activity [10]. Specifically, contact of LSD with the Leu362 residue in TM7 was shown to be seemingly crucial for its agonism and resulting intracellular G-protein or arrestin recruitment [10]. LSD, commonly known as the psychadelic drug “acid” is thought to mediate hallucinogenic “acid trips” through binding to the closely related 5-HT_2A_ serotonin receptor [38]. Therapeutic interest in LSD have emerged from its potential in treating alcoholism, depression, and anxiety in the terminally ill [38]. 5-HT_2B_ and lisuride are also pharmacologically interesting for their role in cardiovascular health: transgenic mice lacking 5-HT_2B_ receptors have heart issues, and this effect is mimicked in normal mice dosed with lisuride [39].

At thermodynamic equilibrium the unliganded 5-HT_2B_ can be conceived of existing in a dynamic equilibrium wherein the accessible conformational states are populated according to the Boltzmann distribution. Binding of a ligand into the OBP or EBP can modify the relative stabilities of the conformational states of the receptor within this ensemble resulting in a rebalancing of the equilibrium population among the various microstates. Within this thermodynamic picture, agonist binding can be thought of as preferentially stabilizing the active state and inducing a thermodynamically favorable driving force for the conformational rearrangement along the activation pathway. Mechanistically, agonist binding induces changes in trigger motifs in the vicinity of the OBP or EBP that facilitate large-scale conformational rearrangements of the transmembrane helices that prime the protein for intracellular G-protein or arrestin binding [10]. Antagonist binding, in contrast, preferentially stabilizes the inactive state and provides no thermodynamic driving force for adoption of the active state.

**Fig 2:**
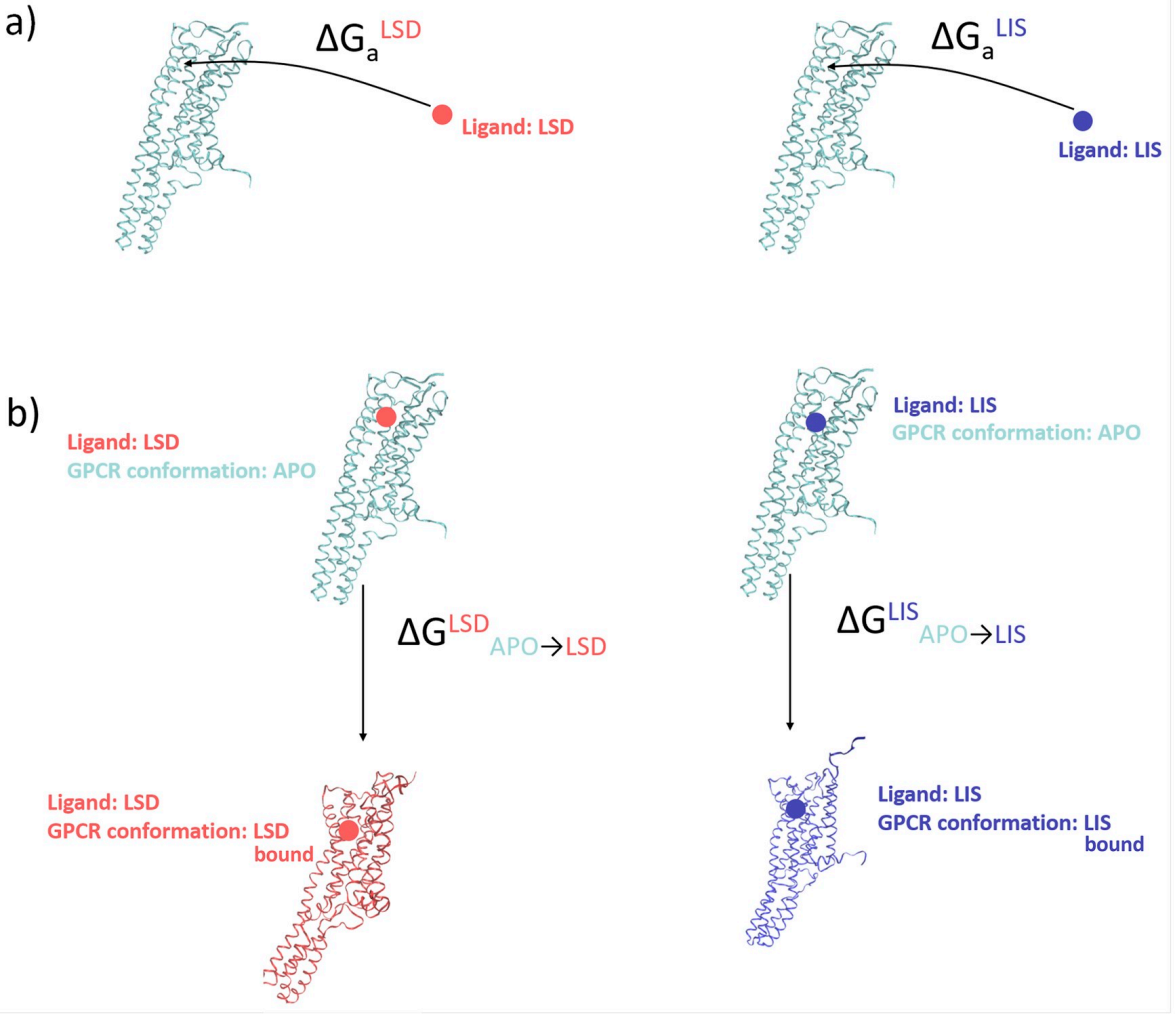
Distinction between the free energy of ligand binding $\Delta G_{a}^{\text{ligand}}$ and free energy of structural relaxation of the receptor-ligand complex with the ligand bound $\Delta G_{\text{APO}\to \text{ligand}}^{\text{ligand}}$. (a) The free energy of ligand binding $\Delta G_{a}^{\text{ligand}}$ is the free energy change associated with the process ligand + receptor (apo) ⇌ ligand–receptor (relaxed) complex, wherein a LSD or lisuride ligand binds to a 5-HT_2B_-TM receptor in the apo conformation and then the ligand–receptor complex relaxes to the stable ligand-bound state. The ligand binding process can be split into two successive steps ligand + receptor (apo) ⇌ ligand–receptor (apo) complex ⇌ ligand–receptor (relaxed) complex. (b) The free energy of structural relaxation $\Delta G_{\text{APO}\to \text{ligand}}^{\text{ligand}}$ corresponds to the free energy change of the second step, wherein the ligand–receptor complex with the 5-HT_2B_-TM receptor in the apo-like conformation relaxes to its thermodynamically stable ligand-bound state. The terminal state and thermodynamic driving force for this relaxation differ for LSD and lisuride ligands.

In this work, we consider an thermostable mutant of 5-HT_2B_ that we term 5-HT_2B_-TM, which has truncated N and C-termini and contains a thermostabilizing replacement of native residues Tyr249-Val313 with Ala1-Leu106 from the thermostabilized apocytochrome b562 RIL [11, 12]. The thermostabilizing mutations restrain the conformational flexibility of the receptor and make it well suited to crystallographic structure determination as reported in Refs. [11, 12]. It is the primary goal of the present work to conduct molecular modeling of the engineered 5-HT_2B_-TM mutant used in crystallography studies, draw comparisons against the experimental structural results, and conduct free energy calculations to shed light on the mechanism of ligand-induced conformational specificity and functional specificity. Our results are primarily of interest in providing deeper thermodynamic and mechanistic understanding of the 5-HT_2B_-TM. The transferability of our results to the wild-type receptor may be assessed by repeating our calculations for the 5-HT_2B_ system. Specifically, we map out changes to the 5-HT_2B_-TM free energy surface in the apo (i.e., unliganded), LSD-bound, and lisuride-bound states. It is our hypothesis that the LSD agonist will stabilize an active-like state of the receptor whereas lisuride will stabilize an inactive-like state, and that these differences can provide the mechanistic basis for the observed functional selectivity [19]. The free energy change $\Delta G_{a}^{\text{ligand}}$ of ligand binding to the 5-HT_2B_-TM receptor is associated with the process,

$$\text{ligand}+{\text{5-HT}}_{\text{2B}}\text{-TM}(\text{apo})⇌\text{ligand}-{\text{5-HT}}_{\text{2B}}\text{-TM}\;(\text{relaxed})\;\text{complex},$$ (1)

wherein a ligand binds to a receptor in the apo conformation and then the ligand–receptor complex relaxes to the stable ligand-bound state (Fig 2a). This thermodynamic process may be (hypothetically) broken into two steps by virtue of free energy being a state function,

$$\text{ligand}+5-{\text{HT}}_{2\text{B}}-\text{TM}(\text{apo})⇌\text{ligand}-5-{\text{HT}}_{2\text{B}}-\text{TM}(\text{apo})\text{complex}$$ (2)

$$⇌\text{ligand}-5-{\text{HT}}_{2\text{B}}-\text{TM}(\text{relaxed})\text{complex},$$ (3)

corresponding first to binding of the ligand into the binding pocket of a 5-HT_2B_-TM receptor in an apo-like conformation and then a subsequent relaxation of the ligand–receptor complex to the stable conformational state by structural rearrangement of the 5-HT_2B_-TM receptor from an apo-like conformation to its thermodynamically stable ligand-bound state. It is the second step that is of principal interest in this work. We term the associated free energy change $\Delta G_{\text{APO}\to \text{ligand}}^{\text{ligand}}$, where the subscript indicates the conformational change from the apo conformation to the stable ligand-bound conformation and the superscript reminds us that this process proceeds with the ligand bound (Fig 2b). The free energy for receptor-ligand binding $\Delta G_{a}^{\text{ligand}}$ is straightforwardly computed from experimental dissociation constants. The free energy for structural relaxation of the ligand-bound receptor $\Delta G_{\text{APO}\to \text{ligand}}^{\text{ligand}}$ is much more challenging to measure experimentally and is computationally estimated in the molecular simulations performed in this work. The structure of the ligand–receptor complex at the end of the thermodynamic relaxation process is of interest in determining whether the stable ligand-bound state is active-like or inactive-like, thereby providing evidence for a mechanistic basis for 5-HT_2B_-TM functional selectivity. The free energy change associated with the structural relaxation provides a measure of the downhill driving force and thermodynamic stability of the relaxed ligand-bound conformation of the receptor relative to the apo state.

It is the primary objective of this work to quantify the perturbations to the conformational free energy surfaces and thermodynamic driving forces for structural relaxation of the ligand–5-HT_2B_-TM complex under LSD and lisuride binding. Since free energy is a state function, the relative stabilities of various configurations are a pure function of state (i.e., independent of history or pathway) and can be measured by estimating the free energy landscape of the receptor mapping out the relative free energy of different receptor configurations. We determine the conformational free energy landscapes of 5-HT_2B_-TM in (i) the unliganded (apo) form, (ii) LSD-bound form, and (iii) lisuride-bound form using all-atom molecular dynamics simulations. We employ well-tempered metadynamics [32–35] to provide good exploration of the conformational free energy landscape for each of the three systems by driving two collective variables quantifying conformational changes in the OBP and resolve the metastable and stable states of each system. The global free energy minimum conformation of the receptor in the LSD–5-HT_2B_-TM complex is structurally similar to the activated state, whereas the global free energy minimum conformation of the receptor in the lisuride–5-HT_2B_-TM complex is structurally similar to the inactive apo state. We employ free energy calculations to drive the ligand-bound complexes between their stable and apo-like conformations to estimate thermodynamic driving forces for structural relaxation of the LSD–5-HT_2B_-TM complex of $\Delta G_{\text{APO}\to \text{LSD}}^{\text{LSD}}\approx -110\;\text{kJ}/\text{mol}$, and of the lisuride–5-HT_2B_-TM complex of $\Delta G_{\text{APO}\to \text{lisuride}}^{\text{lisuride}}\approx -24\;\text{kJ}/\text{mol}$. Our results provide a deeper understanding of the ligand-induced conformational specificity and functional specificity of the 5-HT_2B_-TM seratonin receptor and establish a framework for high-throughput screening or molecular design of pharmaceuticals targeting this physiologically important receptor.

## Materials and methods

### System preparation and relaxation

The unliganded membrane-bound 5-HT_2B_-TM protein (which we henceforward refer to as the APO system for short) was prepared from the LSD-bound crystal structure (PDB: 5TVN) reported in Ref. [11] by removing the ligand from the structure. The 5TVN sequence is an engineered 5-HT_2B_-TM construct that differs from the wild type in lacking a number of N and C-terminal residues and containing thermostabilizing mutations [11]. The protonation state of all residues in the receptor were specified as that corresponding to pH 7 such that the protein carried a (+1) net charge. Protonation states were specified using a classical model of pKa calculations implemented in the ACD Percepta software [40]. We terminated GLU41 with a NH_3_^+^ group and the ARG400 with COO^−^ group using the Gromacs software pdb2gmx and specified residue types HISE for HIS55,242, and HISH for HIS145 and HISD for HIS1064. Both LSD and lisuride were predicted to have pKa of 7.4 for the tertiary amine nitrogen atom that was prepared in its protonated form. The 5-HT_2B_-TM protein was then prepared by Protonation 3D module of MOE software package (v. 2019) developed and distributed by Chemical Computing Group [41], and placed within a 1-palmitoyl-2-oleoyl-sn-glycero-3-phosphocholine (POPC) lipid bilayer comprising 142 molecules in the lower leaflet and 141 in the upper leaflet prepared using MemBuilder [42]. The system was hydrated with water molecules and a single Cl^−^ ion added to maintain charge neutrality [15, 19, 43]. Since we are concerned with a non-biological thermostable mutant of the receptor, we choose not to add additional salt to bring the ionic strength up to physiological levels [43]. The final system dimensions are approximately 9.9 × 9.9 × 15.3 nm^3^ and comprises ∼205,000 atoms. The protein, lipids, and ions were modeled using the Gromos54a7 force field [44] and the water by the SPC model [45].

The LSD-bound (LSD system) and lisuride-bound (LIS system) systems were prepared in an analogous manner to the APO system from the LSD-bound (PDB: 5TVN) and lisuride-bound (PDB: 6DRX) crystal structures reported in Refs. [10, 11] wacker2017crystal, mccorvy 2018structural. The lisuride from 6DRX was transferred into the EBP of the 5TVN protein structure. The LSD ligand was prepared with its tertiary amine nitrogen protonated [11], and parameterized within the semi-empirical QM0 force field using the Automated Topology Builder (ATB) server [46]. The lisuride ligand differs from LSD only in its sterochemistry and an extra NH group, and was prepared in an identical manner. Since both LSD and lisuride carry a (+1) net charge, it was not necessary to add any counterions to these two systems to maintain charge neutrality.

Each of the three systems—APO, LSD, LIS—were then subjected to 1400 steps of steepest descent energy minimization to remove high-energy atomic overlaps, followed by 200 ns of molecular dynamics simulation at at *T* = 323 K and *P* = 1 bar to accelerate structural relaxation. Systems were then cooled to *T* = 310.15 K (body temperature) over the course 100 ns under a linear cooling schedule. Finally, 1.5 *μ*s production runs were conducted at *T* = 310.15 K and *P* = 1 bar. Equations of motion were integrated using the leapfrog algorithm with a time step of 2 fs [47]. Temperature was maintained using a Nosé-Hoover thermostat [48] and pressure maintained using the Parrinello-Rahman barostat [49]. Lennard-Jones interactions were switched smoothly to zero at a 1.2 nm cutoff and dispersion interactions between unlike atoms specified by Lorentz-Berthelot combining rules [50]. Electrostatics treated using particle-mesh Ewald with a real space cutoff of 1.2 nm and a reciprocal space grid spacing of 0.16 nm [51]. Bond lengths were fixed to their equilibrium values using the LINCS algorithm [52]. All calculations were performed using the Gromacs 2018 simulation suite [53]. Our simulation input files are provided as S1 File and have been uploaded to GPCRmd [54] and are locatable at https://submission.gpcrmd.org/view/213/ (APO), https://submission.gpcrmd.org/view/214/ (LSD), and https://submission.gpcrmd.org/view/217/ (LIS).

### Well-tempered metadynamics

Following Provasi *et al*. [19] provasi2011ligand, the conformational free energy surfaces of the APO, LSD, and LIS systems were estimated using well-tempered metadynamics (WTMD) [32–35] to enhance sampling of configurational space and recover an estimate of the molecular free energy landscape. Accelerated sampling is conducted in a small number *k* of user-defined collective variables $s=\left\{s_{i}\left(R\right)\right\}\in R^{k}$ that are themselves a function of the atomic coordinates $R\in R^{3N}$, where *N* is the number of atoms in the system. In brief, this approach lays down a history-dependent bias within the collective variables that drives the simulation to explore previously unvisited regions of configurational space. The metadynamics time-dependent bias potential takes the form of a time-dependent sum of Gaussians,

$$\begin{array}{c} V\left(s\left(R\right),t\right)=\sum_{t^{\prime }<t}\omega_{t^{\prime }}\prod_{i=1}^{k}\exp(-\frac{{\left[s_{i}\left(R\left(t\right)\right)-s_{i}\left(R\left(t^{\prime }\right)\right)\right]}^{2}}{\sigma_{i}^{2}}), \end{array}$$ (4)

where *t*′ is an integer multiple of the deposition time *τ* that dictates the frequency with which Gaussians are deposited, *ω*_*t*′_ controls the height of the Gaussians, and *σ*_*i*_ controls their width. Since we employ well-tempered metadynamics, *ω*_*t*′_ is time dependent and takes the form,

$$\begin{array}{c} \omega_{t^{\prime }}=\omega \exp(-\frac{V(s\left(R\right),t^{\prime })}{k_{B}\Delta T}), \end{array}$$ (5)

where Δ*T* is a parameter with units of temperature, *k*_*B*_ is Boltzmann’s constant, and *ω* is the maximum height of the deposited Gaussians. Δ*T* controls how aggressively the metadynamics bias is applied: in the limit Δ*T* → ∞ we recover standard metadynamics, whereas for Δ*T* = 0 we recover standard molecular dynamics. For this reason it can be convenient to express Δ*T* in terms of the so-called bias factor,

$$\begin{array}{c} \gamma =\frac{T+\Delta T}{T}, \end{array}$$ (6)

where *T* is the temperature of the simulation. As *t* → ∞, the bias potential converges and the free energy landscape in the collective variables can be estimated—up to an arbitrary additive constant—as,

$$\begin{array}{c} F\left(s\left(R\right)\right)=-\frac{T+\Delta T}{\Delta T}V\left(s\left(R\right),t\to \infty \right) \end{array}$$ (7)

The WTMD calculations were conducted using the PLUMED plugin [55] to Gromacs 2018. We adopted as our *k* = 2 collective variables the root mean squared deviation (RMSD) of the OBP (cf. Fig 1b) rotationally and translationally aligned to that of the LSD and LIS systems at the end of the 1.5 *μ*s unbiased simulations. The OBP was defined as the contact residues between LSD ligand and the 5-HT_2B_-TM receptor within the LSD-bound 5-HT_2B_-TM crystal structure (PDB: 5TVN) [11] computed using MSDsite [56]. So defined, the OBP comprises residues ALA130, LEU132, PHE133, ASP135, VAL136, SER139, THR140, ILE186, VAL190, ILE205, CYS207, VAL208, LEU209, PHE217, MET218, GLY221, ALA224, ALA225, TRP337, PHE340, PHE341, ASN344, ILE345, VAL348, LEU362, PHE365, VAL366, GLY369, and TYR370. This pair of collective variables, (RMSD_LIS_, RMSD_LSD_), measures the deviation of the OBP from the lisuride-bound and LSD-bound conformations, and is therefore expected to adequately span the space of configurational motions within the OBP observed in the three systems, resolve metastable states associated with the active and inactive forms of the receptor, and provide interpretable 2D projections of the the free energy surfaces. Clearly this choice of collective variables directly considers only those residues within the OBP, but since ligand-induced perturbations in the OBP are known to induce global conformational changes in the 5-HT_2B_-TM we anticipated that they should also serve as good descriptors of the global protein state. In other words, we make the assumption that under good sampling induced by WTMD, an active (inactive) conformation of the OBP should correspond to an active (inactive) conformation of the entire protein. We demonstrate below that this is indeed the case by verifying that the well-tempered metadynamics calculations achieve convergence, that the free energy landscapes smoothly span the conformational space corresponding to the (meta)stable APO, LSD, and LIS structures, and that the structural changes in key motifs that are indicative of receptor activation are consistent with experimental reports. We observe in passing that more sophisticated approaches to learn good collective variables from the data, as opposed to pre-specifying these in advance, could have been employed [57, 58].

After some preliminary exploration, we found the following choice of parameters to perform well for our WTMD calculations, *σ*_1_ = *σ*_2_ = 0.01 nm, *ω* = 1.0 kJ/mol, and *γ* = 5.0. We obtained converged free energy landscapes spanning ∼120 kJ/mol for each of the three systems over the course of 400 ns production runs. We test for convergence of the simulations by monitoring that there are no longer Gaussians larger than 5% of *ω* being deposited over the course of the terminal 100 ns of the simulation. We also recovered the free energy landscapes within five 20 ns blocks spanning the terminal 100 ns portion of the simulation to verify that the landscapes are converged within this block analysis to better than 0.1 kJ/mol. We define the active structure predicted by the converged WTMD calculations to be the structure occupying the global free minimum of the free energy landscape. Where possible we present a comparison of the computational prediction with available experimental data on the active state. Our PLUMED metadynamics protocol has been uploaded to PLUMED-NEST [59] and is available at https://www.plumed-nest.org/eggs/20/026.

### Umbrella sampling and WHAM

Having recovered estimates of the free energy surfaces parameterized by two collective variables RMSD_LIS_ and RMSD_LSD_ using WTMD, we estimated the thermodynamic driving forces for conformational relaxation of the ligand-receptor complex from an initial apo-like state of the receptor induced by LSD and lisuride binding (Fig 2b). The LIS free energy surface was sufficiently well sampled for us to simply read off the free energy difference $\Delta G_{\text{APO}\to \text{LIS}}^{\text{LIS}}$ between the lisuride-bound global free energy minimum and the coordinates of the global free energy minimum of the APO system (i.e., the lisuride-receptor complex with the receptor in an apo-like configuration). The LSD free energy surface, however, was sufficiently perturbed from the APO landscape that the apo-like configuration was not sufficiently sampled to allow us to read off $\Delta G_{\text{APO}\to \text{LSD}}^{\text{LSD}}$. Accordingly, we employed umbrella sampling (US) and the weighted histogram analysis method (WHAM) to construct a pathway between these states and estimate this quantity [60, 61].

We first identified the (RMSD_LIS_, RMSD_LSD_) coordinates of the global free energy minimum of the LSD system corresponding to the most stable conformation of the LSD-bound 5-HT_2B_-TM receptor. This structure served as the final state for our US calculations. We then identified the (RMSD_LIS_, RMSD_LSD_) coordinates of the global free energy minimum of the APO system corresponding to the most stable configuration of the unliganded 5-HT_2B_-TM. The system configuration residing at these coordinates in the LSD free energy surface corresponds to the apo-like-configuration into which a LSD ligand has been inserted. This serves as the initial state for our US calculations. The free energy difference between the initial and final configurations corresponds to the free energy change $\Delta G_{\text{APO}\to \text{LSD}}^{\text{LSD}}$ associated with the configurational relaxation of the LSD-bound system from an apo-like starting configuration (Fig 2b).

We charted a path between the (RMSD_LIS_, RMSD_LSD_) coordinates of two end states by constructing a series of umbrella windows. As a state function, the free energy difference is independent of the path, so in regions where the LSD free energy landscape determined by WTMD is available, we construct the path to follow its low-free-energy contours for numerical stability. We employ two-dimensional harmonic restraining potentials in the *k* = 2 collective variables (*s*_1_, *s*_2_) = (RMSD_LIS_, RMSD_LSD_),

$$\begin{array}{c} U\left(s\left(R\right)\right)=\sum_{i=1}^{k}\frac{\kappa }{2}{\left(s_{i}\left(R\right)-\xi_{i}\right)}^{2} \end{array}$$ (8)

where *s*_*i*_(**R**) is the value of collective variable *s*_*i*_ corresponding to a system configuration **R**, *κ* is the strength of the restraining potential spring constant, and *ξ* = {*ξ*_1_, *ξ*_2_} is the location of the restraint. We used spring constants of *κ* = 100,000 kJ/mol.nm^2^ and employed 26 umbrella windows. Each umbrella simulation was conducted for 200 ns, and the unbiased potential of mean force (PMF) along the pathway of umbrella windows was reconstructed from the biased simulation trajectories using WHAM [61] implemented in PLUMED [55]. Uncertainties in the calculated free energies are estimated by 100 rounds of bootstrap resampling. The probability distribution functions under each umbrella window rapidly converged and became time invariant on the time scale of tens of ns. An image illustrating adequate overlap between the distribution functions under the umbrella windows is presented in S1 Fig.

The quantities $\Delta G_{\text{APO}\to \text{LIS}}^{\text{LIS}}$ and $\Delta G_{\text{APO}\to \text{LSD}}^{\text{LSD}}$ correspond to the thermodynamic driving forces for structural relaxation of the ligand-receptor complex from the apo-like state of the receptor induced by ligand binding and arise from perturbation of the underlying conformational free energy surface mediated by entry of the ligand into the binding pocket (Fig 2b). We emphasize that the initial and final states of the systems are both ligand-bound, so these free energy differences do not contain any contributions from ligand binding or release.

## Results and discussion

We conducted enhanced sampling molecular dynamics simulations using well-tempered metadynamics to estimate the conformational free energy surfaces for the 5-HT_2B_-TM seratonin receptor in the unliganded (APO system), LSD-bound (LSD system), and lisuride-bound (LIS system) states. Comparison of these free energy landscapes, each of which is parameterized by the RMSD of the OBP residues relative to the lisuride-bound and LSD-bound systems (RMSD_LIS_, RMSD_LSD_), provide a quantitative measure of the perturbation to the conformational free energy landscape of the receptor induced by ligand binding, the driving force for conformational rearrangement of the ligand-receptor complex, and new molecular-level understanding for ligand-induced conformational specificity and functional selectivity.

### Conformational free energy landscapes

**Fig 3:**
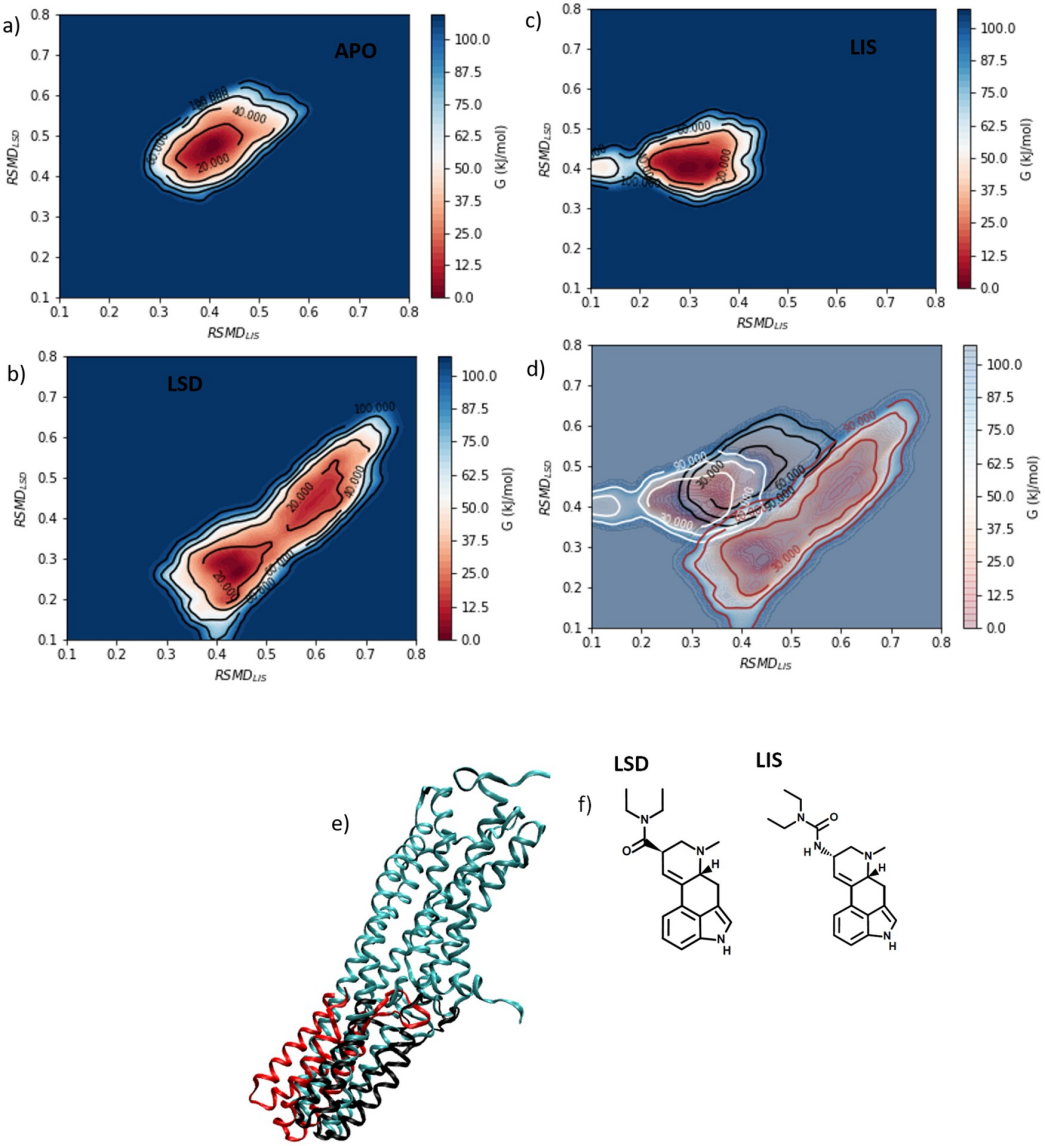
Free energy surfaces in (RMSD_LIS_, RMSD_LSD_) and stable protein conformations computed using well-tempered metadynamics. Free energy surfaces for the three systems a) APO, b) LSD, and c) LIS presented individually, and d) a composite superposition of all three surfaces. The arbitrary additive constant in each free energy landscape was specified by setting the minimum of each free energy landscape to zero. e) The 5-HT_2B_-TM active conformations defined as those residing at the global minimum of each of the three free energy landscapes are presented as aligned and overlaid ribbon structures for the APO (teal), LSD (red), and LIS (black) systems. For viewing clarity we have removed the portions of the receptor lying within the membrane for LSD and LIS systems to better illuminate the large structural differences in the intracellular loops and helices. f) Chemical structures of LSD and lisuride.

We present in Fig 3 the conformational free energy landscapes for the APO, LSD, and LIS systems estimated using well-tempered metadynamics (WTMD) calculations.

#### APO system

The free energy surface for the APO system presented in Fig 3a exhibits a single-well free energy landscape with a deep global minimum at (RMSD_LIS_ = 0.40 nm, RMSD_LSD_ = 0.46 nm) corresponding to the unliganded and inactive form of 5-HT_2B_-TM. We recall that RMSD_LSD_ = 0.0 nm indicates that the OBP precisely matches the conformation of that in the LSD-bound system after 1.5 *μ*s of unbiased simulation, and RMSD_LIS_ = 0.0 nm matches the OBP state in the lisuride-bound system after an equivalent relaxation time. As anticipated, the most stable configuration of the unliganded apo protein is not coincident with either the LSD-bound or lisuride-bound structures. The structure is presented in Fig 3e (teal).

#### LSD system

The LSD system (Fig 3b) exhibits a qualitatively different free energy landscape from the APO system. Relative to APO, the LSD free energy surface is shifted towards lower values of RMSD_LSD_, spans a larger range of RMSD_LIS_, and exhibits two free energy minima in the surface. Our well-tempered metadynamics simulations recover the free energy landscape up to ∼114 kJ/mol above the global free energy minimum, and we observe essentially no overlap between the APO and LSD landscapes within this free energy range in the (RMSD_LIS_, RMSD_LSD_) projection (Fig 3d). The implication of the observed absence of overlap is that low-free energy conformations of the unliganded receptor are high-free energy configurations of the receptor in the LSD–5-HT_2B_-TM complex, and vice versa. This provides a clear indication that binding of the LSD agonist induces large perturbations in the conformational free energy landscape of the 5-HT_2B_-TM receptor and mediates large changes in the distribution of the conformational ensemble of the receptor.

**Fig 4:**
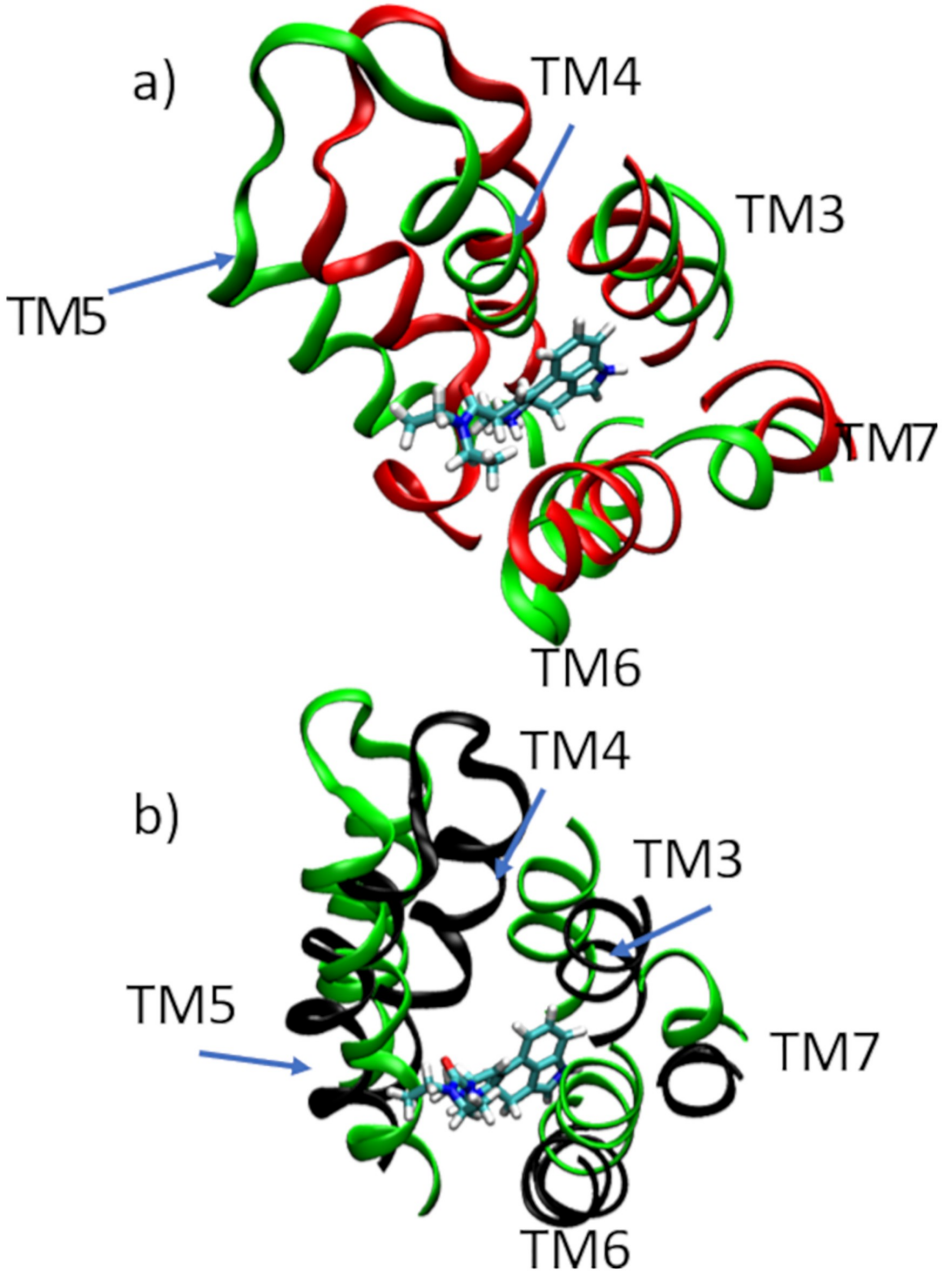
Structural relaxations of the OBP induced by LSD and lisuride binding. a) Comparison of the OBP region of 5-HT_2B_-TM between the global free energy minima of the APO (green ribbon) and LSD (red ribbon) systems. The LSD molecule in the OBP of the LSD system is represented in a licorice rendering. b) Comparison of the OBP region of 5-HT_2B_-TM lisuride the global free energy minima of the APO (green ribbon) and LIS (black ribbon) systems. The lisuride molecule in the OBP of the LIS system is represented in a licorice rendering.

The receptor conformation residing at the global free energy minimum at (RMSD_LIS_ = 0.45 nm, RMSD_LSD_ = 0.25 nm) is structurally close to the LSD-bound crystal structure reported in Ref. [11] (PDB: 5TVN), with a ${\text{RMSD}}_{5\text{TVN}}^{C\alpha }=0.56\;\text{nm}$ computed over all C_*α*_ atoms. We identify this structure as our computational prediction of the active state and refer to it as the active state or stable state for brevity. The structure is presented in Fig 3e (red), and can be seen to differ substantially from the most stable conformation of the APO system (teal) most obviously by a large conformational change in the intracellular helices that move up towards the inner leaflet of the membrane. LSD is a prototypical 5-HT_2B_ agonist [10, 12, 62] and we identify the large conformational change that occurs upon LSD binding as a transition towards an activated state. This is also consistent with prior molecular simulation work that showed structural movement of the intracellular loops and helices associated with binding of the active protein with G protein or arrestin [1, 13, 63, 64]. The conformational change is quantified by comparing the stable LSD structure to the stable APO structure over the TM5-7 regions of the receptor to compute ${\text{RMSD}}_{\text{APO}}^{TM5-7}=0.44\;\text{nm}$. The change in the OBP relative to the stable APO structure upon LSD binding is ${\text{RMSD}}_{\text{APO}}^{OBP}=0.33\;\text{nm}$. The OBP region of the APO and LSD structures are overlaid in Fig 4a. The small change in the OBP upon LSD binding is consistent with the experimental results of McCorvy *et al*. [10] mccorvy2018structural that report similar OBP conformations for a diverse panel of ligands bound to the 5-HT_2B_-TM receptor.

**Fig 5:**
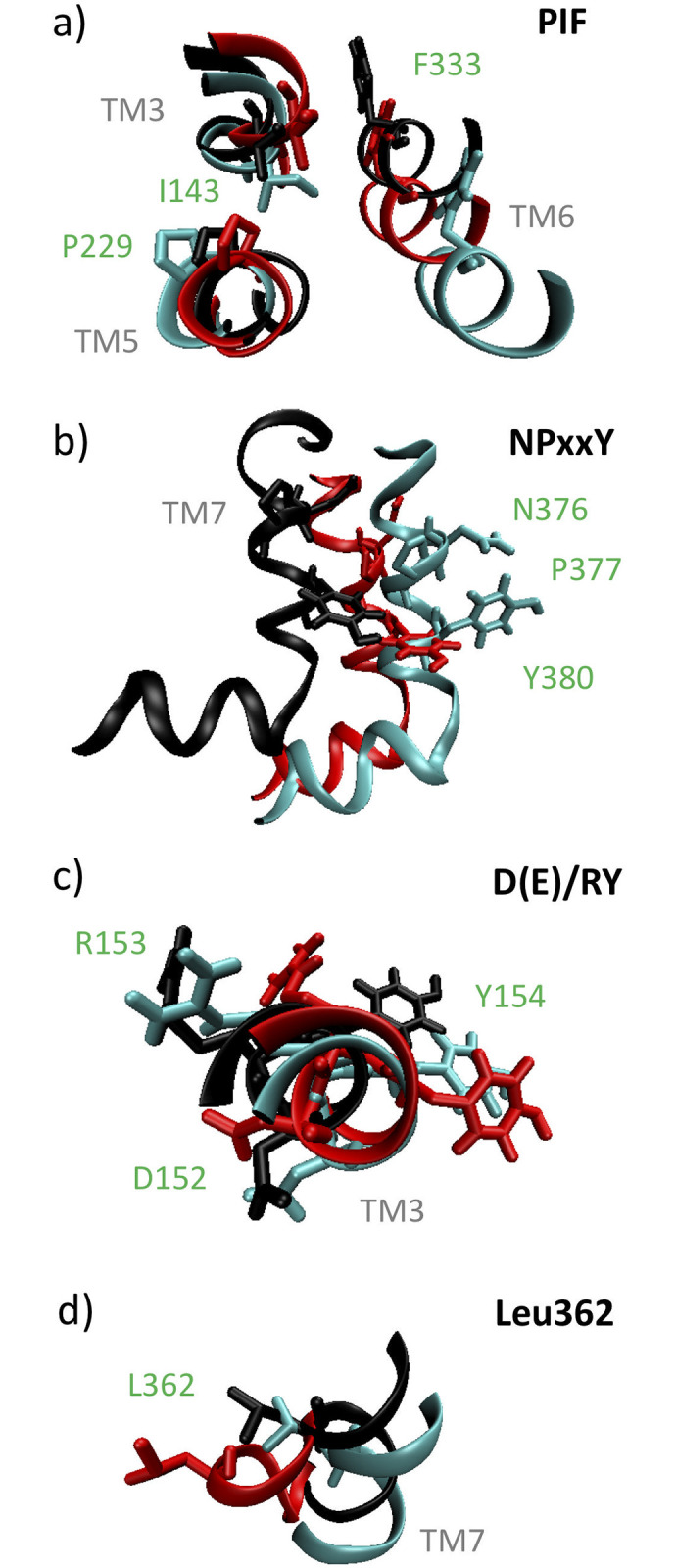
Structural comparison of 5-HT_2B_-TM motifs PIF, NPxxY, D(E)/RY, and Leu362 between the global free energy minima structures of the APO (teal), LSD (red), and LIS (black) systems. a) The PIF motif at the interface of TM3, TM5, and TM6 exhibits inward rotation of the Pro229 on TM5, rotation of the Ile143 on TM3, and displacement of the Phe333 on TM6 consistent with partial activation in both the LSD (red) and LIS (black) systems. b) The NPxxY motif on TM7 exhibits an inward rotation of the Tyr380 and displacement of the TM7 in both the LSD (red) and LIS (black) systems. c) The D(E)/RY motif on TM3 shows relatively little change in terms of the TM3 location and Asp152, Arg153, and Tyr154 side chain positions in the LSD (red) and LIS (black) systems relative to the APO (teal). d) The Leu362 residue in the EBP on TM7 exhibits a large relative displacement in the LSD (red) system compared to the LIS (black).

Wacker *et al*. have previously identified a number of motifs involved in 5-HT_2B_-TM receptor activation [11, 12]. The PIF motif that forms an interface between TM3, TM5, and TM6 was observed to be partially activated in a LSD–5-HT_2B_-TM crystal structure, comprising a shift inward of the TM5 Pro residue, dihedral flip of TM3 Ile, and rotation of the TM6 Phe [11]. Consistent with these findings, relative to the APO structure (teal) the stable LSD structure (red) exhibits an inward rotation of the Pro229, rotation of the Ile143, and a translational shift of the Phe333 and TM6 helix (Fig 5a). The NPxxY motif in TM7 and D(E)/RY motif in TM3 are known as “microswitches” that are implicated in conformational change and priming of the receptor for activation and G-protein binding [8, 12]. The NPxxY motif was observed to be activated in the LSD–5-HT_2B_-TM crystal structure, with marked rotation of the TM7 Tyr into the TM bundle [11]. We find consistency with these experimental findings, with the TM7 and Tyr380 side chain in the stable LSD structure (red) displaced and rotated relative to the APO (teal) (Fig 5b). In the case of the D(E)/RY motif, we observe relatively little change in the TM3 location or relative position of the side chains between the stable LSD (red) and APO (teal) structures (Fig 5c). This is consistent with experimental reports that this motif shows relatively little activation in the LSD–5-HT_2B_-TM crystal structure [11] and that the D152–R153 salt bridge is preserved in its inactive conformation in the ergotamine–5-HT_2B_-TM crystal structure, where ergotamine is the chemical precursor of LSD [12]. We performed these comparisons for the active structures residing at the bottom of the global free energy minima in the APO and LSD conformational free energy landscapes. In S1–S3 Videos we present portions of the WTMD trajectory occupying the global free energy minima to show good structural homogeneity in the PIF, NPxxY, and D(E)/RY motifs and demonstrating that the global free energy minimum structure is representative of the structural ensemble in the vicinity of the global free energy well.

The local free energy minimum at (RMSD_LIS_ = 0.65 nm, RMSD_LSD_ = 0.43 nm) lies Δ*G* ≈ 25 kJ/mol higher in free energy than the global minimum and is substantially closer to the stable APO structure in the TM5-7 regions with a ${\text{RMSD}}_{\text{APO}}^{TM5-7}=0.38\;\text{nm}$. Accordingly, we identify this structure as a more inactive-like LSD-bound structure (Fig 3e (pink)) that is metastable relative to the active-like global minimum (Fig 3e (red)).

Finally, we note that the global free energy minimum of the LSD system lies at RMSD_LSD_ = 0.25 nm, and that conformations at RMSD_LSD_ = 0.0 nm lie more than 100 kJ/mol higher in free energy than the global minimum. Why should configurations of the LSD system that lie closer to RMSD_LSD_ = 0.0 nm be less stable? We recall our definition of our reference state for computation of RMSD_LSD_ as the terminal configuration of the 1.5 *μ*s unbiased simulation of the LSD-bound system. Although this terminal state appeared to be a relaxed stable configuration, the free energy landscapes recovered by WTMD suggest that it was in fact a kinetically trapped configuration that was unable to relax into the global free energy minimum on the time scale of our unbiased calculations. A similar scenario was observed for the LIS system (*vide infra*). These observations are not surprising given the slow time scales associated with GPCR dynamics: passage times of several microseconds for the active to inactive transitions of the *β*_2_AR GPCR have been reported, and passage times for the inactive to active transition occur on still longer time scales and have not been observed in unbiased molecular dynamics simulations [14]. Despite this deficiency in our reference states, (RMSD_LIS_, RMSD_LSD_) still serve as a good discriminatory collective variable in which to conduct the enhanced sampling calculations and parameterize our free energy surfaces. These observations do caution, however, against over-interpreting the structural results of unbiased calculations due to the very slow relaxation times.

#### LIS system

The LIS system (Fig 3c) exhibits a qualitatively similar free energy landscape to the APO system. There is substantial overlap in the APO and LIS landscapes indicating in intersection of the low-free energy structural ensembles (Fig 3d), indicating the binding of the lisuride antagonist causes a relatively mild perturbation to the 5-HT_2B_-TM receptor free energy landscape within the lisuride–5-HT_2B_-TM complex compared to LSD. The global free energy minimum of the LIS system resides at at (RMSD_LIS_ = 0.31 nm, RMSD_LSD_ = 0.41 nm), which is proximate to the APO minimum at (RMSD_LIS_ = 0.40 nm, RMSD_LSD_ = 0.46 nm). Structurally, these two conformations are quite close, with the LIS native structure differing from the APO native structure by only ${\text{RMSD}}_{\text{APO}}^{C\alpha }=0.66\;\text{nm}$ over the *C*_*α*_ atoms. Comparison over the TM5-7 domains returns a relatively large ${\text{RMSD}}_{\text{APO}}^{TM5-7}=0.38\;\text{nm}$, the magnitude of which is ∼85% of that under LSD binding. Comparison of the APO (teal), LSD (red), and LIS (black) stable conformations in Fig 3e shows that the TM5-7 helices in the LIS stable structure continue to point down in a similar pose to the APO structure, such that lisuride binding does not induce TM5-7 to move up and towards the membrane as observed in response to LSD-mediated receptor activation. Lisuride is a prototypical antagonist for 5-HT_2B_-TM and the structure of the LIS stable state supports that it stabilizes an inactive-like state of the receptor. As was observed for LSD, and consistent with McCorvy *et al*. [10], the OBP undergoes only a small change in response to lisuride binding with ${\text{RMSD}}_{\text{APO}}^{OBP}=0.28\;\text{nm}$. The OBP region of the APO and LIS structures are overlaid in Fig 4b.

McCorvy *et al*. determined a lisuride-bound crystal structure and in comparisons to the LSD-bound structure identified similar binding poses of LSD and lisuride within the OBP but different poses in the EBP [10]. This led the authors to propose that the pharmacologically distinct activities of LSD (agonist) and lisuride (antagonist) are mediated through the EBP, and in particular the Leu362 residue on TM7 [10]. Our computational predictions are consistent with these experimental findings, with the stable LSD structure (red) exhibiting a large displacement of the Leu362 residue relative to the APO (teal) compared to a much smaller relative displacement in the stable LIS structure (black) (Fig 5d). We observe similar, and even accentuated, structural changes in the PIF and NPxxY motifs in the stable LIS structures (black) relative to the LSD system (red) that are consistent with (partial) activation (Fig 5a and 5b) [11, 12]. As was the case for the LSD system (red), there is relatively little structural change in the D(E)/RY motif in the stable LIS structure (black) (Fig 5c). A comparison of these motifs between the LIS and LSD systems revealed RMSD_PIF_ = 0.23 nm, RMSD_D(E)/RY_ = 0.16 nm, and RMSD_NPxxY_ = 0.20 nm, indicating small, but significant, changes in the structure of these regions. Experimental characterizations of the structural changes in the PIF, NPxxY, and D(E)/RY motifs in the lisuride-bound complex against which to compare these computational predictions remain to be conducted [10]. S1–S4 Videos show portions of the WTMD trajectory within the global free energy minima and illustrate good structural homogeneity in the PIF, NPxxY, D(E)/RY, and Leu362 motifs.

The overlap between the APO and LIS free energy landscapes allows us to read-off the free energy difference between the coordinates of the APO (RMSD_LIS_ = 0.40 nm, RMSD_LSD_ = 0.46 nm) and LIS (RMSD_LIS_ = 0.31 nm, RMSD_LSD_ = 0.41 nm) minima in the APO and LIS systems. In the APO system, this corresponds to the free energy cost associated with changing the conformation of the unliganded receptor from its global free energy minimum to a structure resembling the native structure of the lisuride-bound system *in the absence of the ligand*. We term this quantity $\Delta G_{\text{APO}\to \text{LIS}}^{\text{APO}}$. In the LIS system, this corresponds to the free energy cost associated with changing the conformation of the lisuride-bound system from its global free energy minimum to a structure resembling the native structure of the unliganded system *with the lisuride ligand bound*. We term this quantity $\Delta G_{\text{LIS}\to \text{APO}}^{\text{LIS}}$. Interrogation of our APO free energy landscape reveals $\Delta G_{\text{APO}\to \text{LIS}}^{\text{APO}}=(38.4\pm 6.0)\;\text{kJ}/\text{mol}$, indicating that the lisuride-bound-like conformation is a somewhat low-lying member of the unliganded conformational ensemble. Uncertainties are estimated by 100 bootstrap resamples. Similarly, interrogation of our LIS free energy landscape reveals $\Delta G_{\text{LIS}\to \text{APO}}^{\text{LIS}}=(23.6\pm 5.0)\;\text{kJ}/\text{mol}$, indicating that unliganded-like conformation is a relatively low-lying member of the lisuride-bound conformational ensemble.

The quantity $\Delta G_{\text{APO}\to \text{LIS}}^{\text{LIS}}=(-\Delta G_{\text{LIS}\to \text{APO}}^{\text{LIS}})=-(23.6\pm 5.0)\;\text{kJ}/\text{mol}$ can be interpreted as the favorable thermodynamic driving force for conformational relaxation of the lisuride–5-HT_2B_-TM complex by structural rearrangement of the lisuride-bound 5-HT_2B_-TM receptor from an apo-like conformation to its thermodynamically stable lisuride-bound state. Physically, this process can be conceived of as the reversible work associated with the conformational relaxation of the 5-HT_2B_-TM receptor after inserting the lisuride ligand into the binding pocket of the apo conformation (Fig 2b). The small value of this quantity is consistent with the relatively minor nature of the perturbation to the 5-HT_2B_-TM free energy landscape induced by lisuride binding.

### Ligand-induced conformational rearrangement free energies

**Fig 6:**
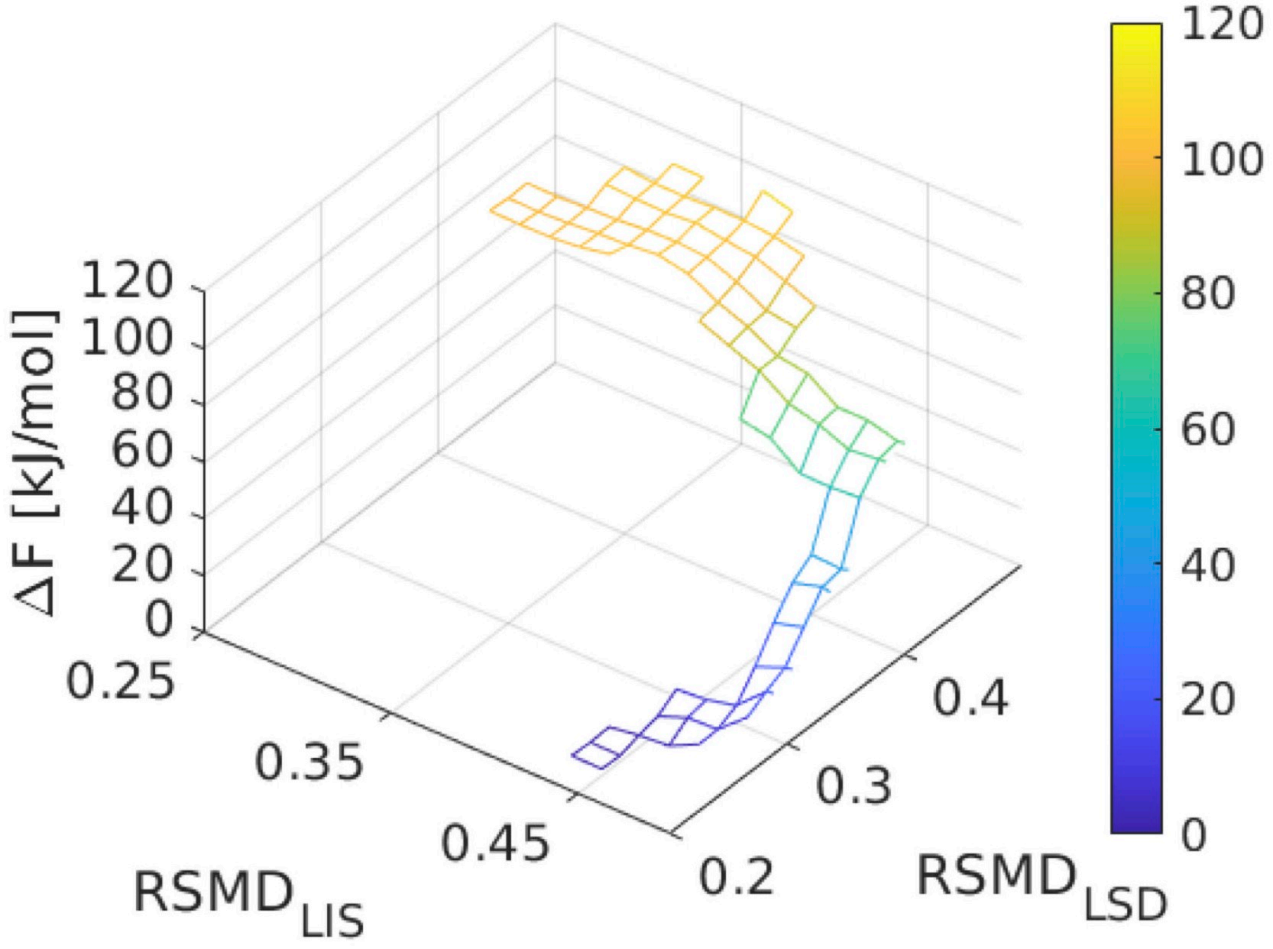
Free energy pathways connecting the LSD global free energy minima with the apo-like state. Umbrella sampling free energy pathway connecting the global minimum of the LSD system (RMSD_LIS_ = 0.45 nm, RMSD_LSD_ = 0.25 nm) to the position of the global minimum of the APO system (RMSD_LIS_ = 0.40 nm, RMSD_LSD_ = 0.46 nm). The downhill free energy change corresponds to the reversible work associated with conformational relaxation of the LSD–5-HT_2B_-TM complex by structural rearrangement of the LSD-bound 5-HT_2B_-TM receptor from an apo-like conformation to its thermodynamically stable LSD-bound state.

In the previous section, we reported $\Delta G_{\text{APO}\to \text{LIS}}^{\text{LIS}}$ by reading this off the LIS free energy landscape. Due to the disjoint nature of the APO and LSD free energy landscapes from WTMD (Fig 3d) we are unable to read off an analogous quantity $\Delta G_{\text{APO}\to \text{LSD}}^{\text{LSD}}$ for LSD. Instead, we employed umbrella sampling to construct a path in the LSD system between the coordinates of the global minimum in the LSD system (RMSD_LIS_ = 0.45 nm, RMSD_LSD_ = 0.25 nm) and the global minimum of the APO system (RMSD_LIS_ = 0.40 nm, RMSD_LSD_ = 0.46 nm). We illustrate in Fig 6 the free energy pathway constructed between these states using umbrella sampling, which provides an estimate of $\Delta G_{\text{APO}\to \text{LSD}}^{\text{LSD}}=-(110.3\pm 0.4)\;\text{kJ}/\text{mol}$. This is the favorable thermodynamic driving force for conformational relaxation of the LSD–5-HT_2B_-TM complex by structural rearrangement of the LSD-bound 5-HT_2B_-TM receptor from an apo-like conformation to its thermodynamically stable LSD-bound state. Physically, this quantity can be interpreted as the reversible work associated with the conformational relaxation of the 5-HT_2B_-TM receptor after inserting the LSD ligand into the binding pocket of the apo conformation (Fig 2b).

The value of $\Delta G_{\text{APO}\to \text{LSD}}^{\text{LSD}}=-(110.3\pm 0.4)\;\text{kJ}/\text{mol}$ is approximately five times larger than $\Delta G_{\text{APO}\to \text{LIS}}^{\text{LIS}}=-(23.6\pm 5.0)\;\text{kJ}/\text{mol}$. This reflects the much larger perturbation of the 5-HT_2B_-TM conformational free energy landscape induced by the LSD agonist compared to the lisuride antagonist, and a much greater thermodynamic gradient for conformational rearrangement ultimately leading to receptor activation. The driving force for conformational relaxation of the lisuride–5-HT_2B_-TM complex is the same order of magnitude as the values reported by Provasi *et al*. for the conformational relaxation of *β*_2_AR bound to a full agonist, weak partial agonist, two inverse agonists, and one neutral agonist [19]. Our calculated value for LSD binding is approximately ten times larger, indicating a far greater driving force for the LSD-induced activation of 5-HT_2B_-TM than any of the *β*_2_AR agonists explored in that work.

It is also instructive to compare our calculated free energies for structural relaxation of the ligand-receptor complex ($\Delta G_{\text{APO}\to \text{ligand}}^{\text{ligand}}$, Fig 2b) with the experimentally determined ligand binding free energies ($\Delta G_{a}^{\text{ligand}}$, Fig 2a). Millan *et al*. report an experimental dissociation constant for lisuride binding at *T* = 295 K of *pK*_*d*_ = -log_10_ *K*_*d*_ = 9.87 [65], from which we compute a strongly favorable standard free energy of association—at a standard reference concentration of *c*^⦵^ = 1 M—of $\Delta G_{a}^{\text{LIS}}=RT\;\text{ln}\;K_{d}=-55.7\;\text{kJ}/\text{mol}$. McCorvy *et al*. [10] report an experimental dissociation constant for LSD binding at *T* = 310 K of *pK*_*d*_ = 9.33, from which we compute a very similar standard free energy of association of $\Delta G_{a}^{\text{LSD}}=RT\;\text{ln}\;K_{d}=-55.4\;\text{kJ}/\text{mol}$. The calculated standard binding free energies are about two times larger than the conformational rearrangement free energy upon lisuride binding $\Delta G_{\text{APO}\to \text{LIS}}^{\text{LIS}}=-(23.6\pm 5.0)\;\text{kJ}/\text{mol}$, and about two times smaller than the conformational rearrangement free energy upon LSD binding $\Delta G_{\text{APO}\to \text{LSD}}^{\text{LSD}}=-(110.3\pm 0.4)\;\text{kJ}/\text{mol}$.

## Conclusions

In this work, we have reported the use of enhanced sampling molecular dynamics simulations to determine the conformational free energy landscapes of the 5-HT_2B_-TM thermostable mutant of the 5-HT_2B_ seratonin receptor in the apo state, LSD-bound state, and lisuride-bound state. LSD is a prototypical agonist for 5-HT_2B_, and its binding induces a large perturbation to the conformational free energy landscape of the receptor within the ligand-receptor complex to the degree that the structural ensembles of the apo and LSD-bound states become effectively disjoint. LSD binding shifts the global minimum of the ligand-receptor complex free energy landscape to the active state and induces a thermodynamic driving force for structural activation of Δ*G* ≈ -110 kJ/mol. We also observe the presence of a metastable inactive-like LSD-bound structure that lies Δ*G* ≈ 25 kJ/mol higher in free energy. Lisuride, on the other hand, is a prototypical antagonist and its binding induces a relatively smaller perturbation of the conformational free energy landscape. The structural ensembles for the apo and lisuride-bound states show a high degree of similarity, and the global minimum of the lisuride-bound free energy landscape shows close structural similarity with the inactive apo form and lies only Δ*G* ≈ 24 kJ/mol lower in free energy. Our work also reveals that the structural conformations adopted by long 1.5 *μ*s unbiased molecular dynamics simulations do not correspond to the most stable structures identified in our enhanced sampling calculations, revealing the value of accelerated sampling and cautioning against over interpreting unbiased molecular simulations due to the long relaxation time scales associated with these systems.

Our results quantify the driving forces for activation of 5-HT_2B_-TM by LSD binding and demonstrate the absence of this driving force under lisuride binding, thereby shedding light on the molecular-level structural and thermodynamic basis for ligand-induced conformational specificity and functional selectivity. Future work may consider the binding of other drugs and ligands and the introduction of path sampling techniques such as forward flux sampling to quantify the kinetics of the conformational rearrangement events [66, 67]. This work also establishes a framework for high-throughput virtual screening of putative 5-HT_2B_ ligands, and a principled platform for drug design through the rational engineering of ligand-bound free energy landscapes [19, 68].

## Supporting information

S1 File Simulation input files. Zip archive containing input files for simulation of the APO, LSD, and LIS systems.
(ZIP)

S1 Fig Umbrella sampling histograms. The probability density functions under each of the umbrella sampling windows show good overlap between windows and form a well sampled path between the two end states.
(TIF)

S1 Video PIF movie. Movie showing 56 ns trajectory of PIF motif for aligned active (i.e., global free energy minimum) structures of the APO (teal), LSD (red), and LIS (black) systems. Frames are rendered every 100 ps.
(MPG)

S2 Video NPxxY movie. Movie showing 56 ns trajectory of NPxxY motif for aligned active (i.e., global free energy minimum) structures of the APO (teal), LSD (red), and LIS (black) systems. Frames are rendered every 100 ps.
(MPG)

S3 Video D(E)/RY movie. Movie showing 56 ns trajectory of D(E)/RY motif for aligned active (i.e., global free energy minimum) structures of the APO (teal), LSD (red), and LIS (black) systems. Frames are rendered every 100 ps.
(MPG)

S4 Video Leu362 movie. Movie showing 56 ns trajectory of Leu362 motif for aligned active (i.e., global free energy minimum) structures of the APO (teal), LSD (red), and LIS (black) systems. Frames are rendered every 100 ps.
(MPG)
