# Supplementary figures and images for: Free energy calculations of the functional selectivity of 5-HT2B G protein-coupled receptor
Source: PLoS One. 2020 Dec 9;15(12):e0243313. doi: 10.1371/journal.pone.0243313 (PMC7725398; doi:10.1371/journal.pone.0243313)

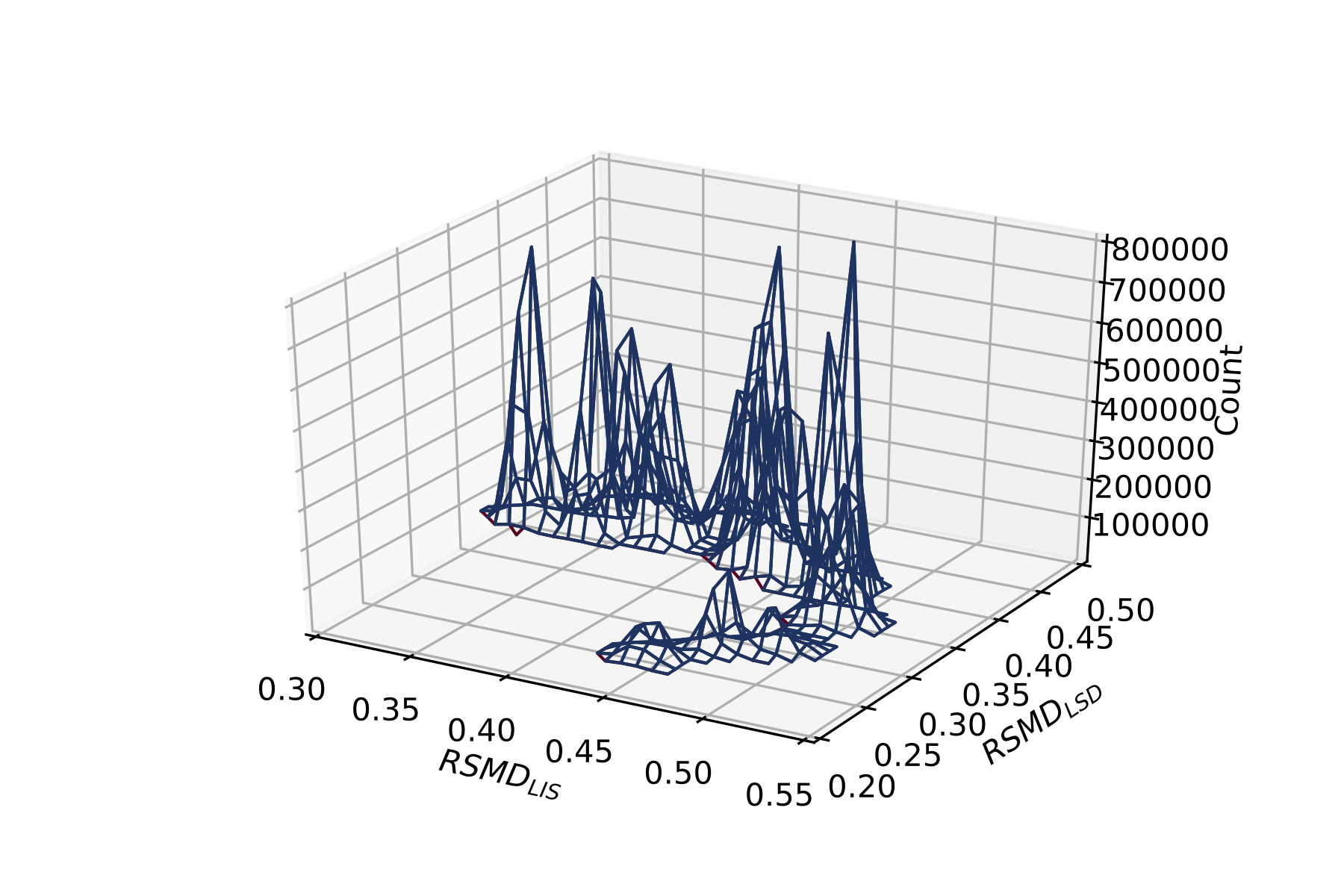

Supplement: S1 Fig — The probability density functions under each of the umbrella sampling windows show good overlap between windows and form a well sampled path between the two end states. (TIF) [file pone.0243313.s002.tif]
